# Supplementary material for: Netcombin: An algorithm for constructing optimal phylogenetic network from rooted triplets
Source: PLoS One. 2020 Sep 18;15(9):e0227842. doi: 10.1371/journal.pone.0227842 (PMC7500971; doi:10.1371/journal.pone.0227842)
Supplement: S4 File — (ZIP) [file pone.0227842.s004.zip › SequenceData-Triplet/sequenceData-10-20-40-Triplet2/data-(sample 10- length 500- num seq 20-m rate 10^-6)/6 seq.pdf]

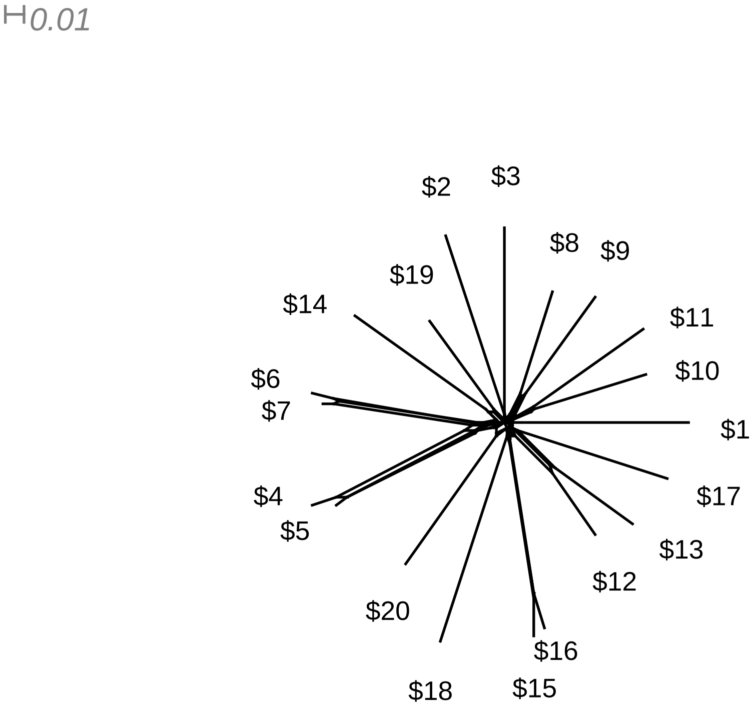

0.01
$2
$3
$8
$9
$19
$14
$11
$10
$6
$7
$1
$17
$4
$5
$13
$12
$20
$16
$18
$15
